# The impact of mindfulness on nurses’ perceived professional benefits: the mediating roles of workplace spirituality and work-life balance

**DOI:** 10.3389/fpsyg.2024.1346326

**Published:** 2024-02-27

**Authors:** Yapeng Lin, Changchun Jiang, Yujing Pan, Ziqing Xu

**Affiliations:** ^1^School of Management, Putian University, Putian, China; ^2^School of Performance and Cultural Industries, University of Leeds, Leeds, United Kingdom; ^3^Business College, Guangdong Ocean University, Yangjiang, China

**Keywords:** mindfulness, workplace spirituality, perceived professional benefits, work-life balance, nurses

## Abstract

This study delves into the effects of mindfulness on workplace spirituality, work-life balance, and perceived professional benefits among nurses operating in the high-pressure environments of hospitals in Jiangxi Province, China. Utilizing a robust sample of 303 valid questionnaires and employing partial least squares (PLS) analysis, the research uncovers a significant positive relationship between mindfulness and workplace spirituality. Furthermore, it demonstrates how both workplace spirituality and work-life balance serve as crucial mediators in enhancing nurses’ perception of their professional benefits. The findings illuminate the potential of mindfulness training in substantially elevating job satisfaction and reducing burnout among nurses. The study not only reinforces the value of mindfulness in the healthcare sector but also advocates for its integration into professional development programs and healthcare policies. By doing so, it aims to bolster the overall wellbeing and professional effectiveness of nurses facing the myriad challenges inherent in demanding healthcare environments. This study contributes to the growing discourse on mindfulness in occupational settings, highlighting its pivotal role in enhancing both the personal wellbeing and professional capabilities of healthcare professionals.

## 1 Introduction

In the demanding realm of healthcare, nurses emerge as the linchpin, directly influencing patient outcomes and shaping the trajectory of care. These healthcare professionals, by virtue of their roles, are constantly navigating a plethora of challenges that span both the clinical and emotional dimensions of care (Huang et al., 2020; Wu et al., 2020). Beyond the immediate medical responsibilities, the emotional and psychological facets of nursing often remain overshadowed, despite their profound impact on overall wellbeing (Vermaak et al., 2017). The intricate balance between providing empathetic patient care and managing their own emotional wellbeing places nurses in a unique position, making them susceptible to burnout and job dissatisfaction (Salvarani et al., 2020). Literature has consistently highlighted the heightened stress levels and burnout rates among nurses, emphasizing the need for interventions and strategies to enhance their wellbeing (Khamisa et al., 2015; Bakhamis et al., 2019). Amidst this backdrop of professional demands and the quest for personal equilibrium, the potential of mindfulness in enhancing nurses’ perceived professional benefits takes center stage. Mindfulness, characterized by a deliberate focus and presence in the moment, has been gaining traction for its potential benefits in various professional settings, including healthcare (Garland et al., 2015; Eby et al., 2020). Rooted in ancient practices, mindfulness offers a contemporary solution, providing individuals a mechanism to navigate their professional roles with greater clarity, resilience, and balance. The emphasis on mindfulness in this study is not just a trend but a response to the pressing need for tools that can positively influence wellbeing and job satisfaction, especially in high-stress professions like nursing (Levett et al., 2019; Piotrowski et al., 2022).

Previous studies have found various factors that contribute to the wellbeing and job satisfaction of nurses, such as supportive work environments (Smith, 2022), effective communication skills (Kirca and Bademli, 2019), and adequate work-life balance (Schluter et al., 2011). Additionally, aspects like emotional intelligence (Christianson, 2020) and resilience (Delgado et al., 2017) have been identified as key elements that help nurses manage the challenges inherent in their profession. While these studies provide valuable insights, there remains a gap in understanding the specific impact of mindfulness on nurses’ perceived professional benefits, particularly through the mediating roles of workplace spirituality and work-life balance. Although mindfulness has been recognized for its positive effects in various professional settings (Roeser et al., 2012; Zheng et al., 2020), its direct influence on the nursing profession, especially in high-pressure healthcare environments, is not extensively explored. Furthermore, the interplay between mindfulness, workplace spirituality, and work-life balance in shaping nurses’ professional experiences and benefits is an area that has received limited attention in existing literature. This study aims to fill this research gap by examining how mindfulness practices among nurses can enhance their perceived professional benefits, with a focus on the mediating roles of workplace spirituality and work-life balance. By doing so, this research not only contributes to the broader discourse on mindfulness in occupational settings but also offers specific insights into its application and benefits in the nursing field.

Mindfulness is often described as a flexible state of mind that enables individuals to focus attentively on the present moment (Wheeler et al., 2017), and its potential benefits in various professional settings are increasingly recognized. Brown and Ryan (2003) view mindfulness as a personality trait. While the business and management fields have extensively studied trait mindfulness, significant research gaps remain in the nursing field, particularly regarding how mindfulness, through the mediating roles of workplace spirituality and work-life balance, impacts nurses’ perceived professional benefits. Existing research has primarily focused on the impact of mindfulness on stress management (Chen and Murphy, 2019; Charoensukmongkol and Puyod, 2022). However, there is less development in exploring how mindfulness affects specific professional outcomes, such as job performance and satisfaction, especially in high-pressure professions like nursing. Recent studies have begun to investigate the positive effects of trait mindfulness on work performance in various environments (King and Haar, 2017; Hafenbrack and Vohs, 2018), but exploration within the nursing field, particularly regarding the mediating roles of workplace spirituality and work-life balance, remains limited. This study aims to fill this gap by examining how mindfulness as a trait can enhance nurses’ perceived professional benefits through the enhancement of workplace spirituality and work-life balance. It seeks to understand not only the direct impact of mindfulness on professional outcomes but also how it indirectly influences these outcomes through these mediating factors. Moreover, this study will explore the process through which mindfulness benefits nurses in their professional roles. If mindfulness is indeed crucial for improving nurses’ professional experiences, could its effects be mediated by certain aspects of their work life that are enhanced by mindfulness? This study will address these gaps by exploring how specific aspects of the nursing profession might influence the effectiveness of mindfulness in improving job satisfaction and overall professional wellbeing.

This study primarily aims to address the identified research gaps in the context of nursing. Firstly, it examines whether nurses who exhibit high levels of mindfulness experience greater perceived professional benefits compared to those who do not demonstrate this trait. Additionally, the study explores the potential mediating roles of workplace spirituality and work-life balance in this relationship. Given their relevance to the nursing profession, workplace spirituality (including a deep connection with work and a sense of purpose) and work-life balance (focusing on harmonizing professional and personal life) are selected as mediating variables (Foster and Foster, 2019; Zhang, 2020; Pattanawit and Charoensukmongkol, 2022). These factors are crucial in high-pressure healthcare environments, as nurses often face challenges in maintaining personal wellbeing while delivering quality patient care (Siffleet et al., 2015). Despite the evident importance of workplace spirituality and work-life balance, not all nurses actively engage in practices that promote these aspects. Therefore, this study proposes that mindfulness can significantly contribute to enhancing workplace spirituality and work-life balance. Enhanced mindfulness can heighten nurses’ awareness of their work environment and personal needs, thereby helping them recognize the value of integrating these aspects into their professional lives. Furthermore, the healthcare environment, characterized by high stress and emotional demands, reflects the level of challenges faced by nurses daily. Nurses with a high degree of trait mindfulness are likely to be more aware of these challenges, which may encourage them to more actively engage in practices that enhance workplace spirituality and work-life balance, ultimately leading to an increase in perceived professional benefits.

This study extends previous research in two key areas. Firstly, it explores how trait mindfulness enhances nurses’ perceived professional benefits through the mediating roles of workplace spirituality and work-life balance. This aspect is crucial for understanding the specific application and effectiveness of mindfulness in the nursing profession, particularly within high-pressure healthcare environments. Secondly, the study examines how workplace spirituality and work-life balance act as vital mediating variables, linking mindfulness with nurses’ job satisfaction and wellbeing. This approach not only reveals the direct impact of mindfulness on nurses’ professional lives but also unveils the mechanism through which it indirectly influences professional outcomes via these mediating factors. The study is expected to provide new insights into the application of mindfulness in the field of nursing, especially regarding how it can be effectively utilized to enhance nurses’ professional wellbeing and job satisfaction. The anticipated results are expected to offer strategic recommendations for healthcare institutions to foster the professional development and personal wellbeing of nurses, particularly in managing high-pressure work environments. The following sections will discuss the importance of trait mindfulness and how it impacts workplace spirituality and work-life balance, thereby improving nurses’ professional performance and perceived professional benefits.

The following sections are structured as follows: section “2 Literature review” presents the hypotheses and conceptual models. Section “3 Materials and methods” introduces data collection and data analysis methods. Section “4 Result” describes the results of the data analysis and tests the hypotheses. Section “5 Discusses” the theoretical implications, practical implications, and limitations with directions for future studies. Section “6 Conclusion” has a short.

## 2 Literature review

### 2.1 Mindfulness

Mindfulness, as conceptualized in contemporary literature, is often described as “paying attention in a particular way: on purpose, in the present moment, and non-judgmentally” (Kabat-Zinn, 1994). As an inherent trait, it signifies an individual’s propensity to remain anchored in the present, actively observing experiences without being overly reactive or overwhelmed by them. Central to trait mindfulness are the elements of focused attention and heightened awareness (Brown and Ryan, 2003). Individuals with pronounced mindfulness traits exhibit enhanced self-regulation, making them adept at navigating complex emotional landscapes without succumbing to impulsive behaviors (Baer et al., 2004; Reb et al., 2014). Mindful individuals possess the unique ability to filter distractions, both internal and external, thereby sustaining concentration on the task at hand (Bishop et al., 2004). Their capacity for objective evaluation is also noteworthy, enabling them to process stimuli without the interference of preconceived notions or biases (Teper et al., 2013). This objectivity often shields them from forming hasty judgments, allowing for more measured and thoughtful responses.

Recent empirical investigations underscore the myriad benefits of trait mindfulness. It has been linked to enhanced psychological wellbeing, fostering resilience, and bolstering interpersonal dynamics (Davidson et al., 2003). In the realm of professional decision-making, mindfulness has been spotlighted for its role in refining managerial choices (Reb et al., 2014) and guiding investors in adhering to strategic financial plans (Kudesia, 2019). Moreover, the positive impact of mindfulness on task performance, especially in tasks demanding cognitive rigor, has been well-documented (Dane, 2011). Research by Jha et al. (2007) further elucidates how mindfulness augments cognitive regulation, optimizing performance in novel and intricate tasks. Additionally, findings from Zeidan et al. (2010) corroborate that individuals with heightened mindfulness exhibit superior outcomes in tasks of complexity.

### 2.2 Cognitive resource theory

Cognitive resource theory, initially proposed by Fiedler (1989), offers a framework for understanding how cognitive abilities and situational factors interplay to influence individual performance. The theory posits that performance outcomes are contingent on the alignment of cognitive resources with task demands and environmental complexities. It emphasizes that while cognitive abilities are crucial in complex and demanding situations, their effectiveness is moderated by factors such as stress levels, task nature, and individual experiences (Vecchio, 1990; Murphy et al., 1992). In the nursing profession, cognitive resource theory becomes particularly relevant due to the diverse and often challenging nature of nursing tasks. Nurses are required to make quick, accurate decisions in high-pressure environments, where cognitive abilities are continuously tested. The theory suggests that in such complex scenarios, enhanced cognitive resources, such as those developed through mindfulness practices, are vital for effective performance. Mindfulness, characterized by an intentional and non-judgmental focus on the present moment (Kabat-Zinn, 1990), is posited to enhance cognitive resources. It improves attention, awareness, and clarity, enabling nurses to better navigate the complexities of their work. Recent studies have shown that mindfulness practices can reduce cognitive overload and improve decision-making abilities, aligning well with the cognitive resource theory’s emphasis on the importance of cognitive abilities in complex tasks (Hölzel et al., 2011; Lutz et al., 2016).

In summary, the cognitive resource theory provides a valuable lens for examining the impact of mindfulness on nurses’ performance and wellbeing. By enhancing cognitive resources, mindfulness not only directly influences nurses’ ability to handle complex tasks but also indirectly affects their job satisfaction and wellbeing through the mediating roles of workplace spirituality and work-life balance. This theory underscores the importance of mindfulness in nursing, suggesting that its practice can be a key strategy for enhancing cognitive resources and, consequently, professional benefits in nursing.

### 2.3 Effect of mindfulness on workplace spirituality and work-life balance

Spirituality at workplace is as simple as having a sense of connection between own self and the workplace (Rathee and Rajain, 2020). Workplace spirituality is a concept that involves experiencing a deep sense of personal fulfillment and meaningful connection within one’s work environment. It encompasses elements such as a profound connection to one’s work, alignment with the organization’s values, and a sense of purpose and contribution to a greater good (Jnaneswar and Sulphey, 2021). This concept goes beyond mere job satisfaction, focusing instead on how individuals integrate their personal values and beliefs with their professional lives, creating a harmonious and fulfilling work experience.

Kabat-Zinn (1990) defines mindfulness as the act of paying attention in a particular way, intentionally, in the present moment, and non-judgmentally. A salient feature of mindfulness that bolsters workplace spirituality is its ability to cultivate a heightened sense of awareness and connection to one’s surroundings (Brown and Ryan, 2003). This heightened state of awareness can deeply resonate with the core tenets of workplace spirituality, as it enables individuals to connect more profoundly with their work and find greater meaning in their professional roles.

Given that mindful individuals are more attuned to their experiences and can process them without being overly reactive, they are better positioned to recognize and align with the spiritual dimensions of their work (Dane, 2011; Rehman and Dastgeer, 2022). This alignment has been corroborated in previous studies. For instance, Good et al. (2016) discovered that individuals who practiced mindfulness reported a stronger sense of purpose and connection in their roles. Moreover, Bamber and Schneider (2022) assert that a mindful individual, by being fully engaged in their tasks and interactions, can better perceive and resonate with the spiritual undertones of their profession. Additionally, Liu et al. (2020) demonstrated that mindfulness practices could foster a deeper understanding and appreciation of one’s role, thereby amplifying the spiritual dimensions of work.

Given the profound influence of mindfulness in nurturing and enhancing the spiritual aspects of one’s profession, especially in a demanding and emotionally charged environment like nursing, the following hypothesis is presented:

**Hypothesis 1 (H1):** The practice of mindfulness by nurses will significantly enhance their sense of workplace spirituality.

Work-life balance refers to the state of equilibrium where a person equally prioritizes the demands of one’s career and the demands of one’s personal life. It involves managing the juggling act between work, family, leisure, and other personal activities, aiming for an optimal distribution of an individual’s time and focus across these various aspects of life (Onu et al., 2018). Althammer et al. (2021) argue that work-life balance is not merely about the equal distribution of time between work and personal life, but more about the psychological equilibrium and satisfaction derived from both domains.

Mindfulness, as conceptualized by McNall et al. (2021), emphasizes the importance of being fully present in the moment, engaging with current experiences without judgment. By being fully present, individuals can better assess their current needs, both professionally and personally, leading to more informed decisions about time allocation and prioritization (Thimmapuram et al., 2019; Mellner et al., 2022). This heightened awareness can also reduce the cognitive rumination about work during personal time, and vice versa, thereby enhancing the quality of experiences in both domains (Yang and Jo, 2022).

Prior research has provided empirical evidence supporting the role of mindfulness in promoting work-life balance. For instance, Pacheco et al. (2021) found that individuals practicing mindfulness reported lower work-life conflict and greater satisfaction in both work and personal life domains. Similarly, Daniel et al. (2022) demonstrated that mindfulness practices can reduce the negative spillover effects of work stress into personal life, thereby fostering a more harmonious work-life integration. Furthermore, Slutsky et al. (2019) posited that by cultivating a mindful approach, individuals can develop greater resilience and flexibility, essential traits for navigating the demands and challenges of balancing work and personal life.

Given the potential of mindfulness in enhancing the equilibrium between professional and personal life, the following hypothesis is presented:

**Hypothesis 2 (H2):** The practice of mindfulness by nurses will significantly enhance their work-life balance.

### 2.4 Effect of workplace spirituality and work-life balance on perceived professional benefits

Workplace spirituality is defined as the recognition and integration of employees’ spiritual beliefs within the work environment, fostering a sense of purpose, interconnectedness, and alignment with organizational values (Indradevi, 2020). This concept transcends traditional job satisfaction metrics, emphasizing a deeper, more intrinsic form of fulfillment derived from work.

In the nursing profession, workplace spirituality is particularly important due to the emotionally and spiritually demanding nature of the job. Nurses often seek more than just financial rewards or career progression; they look for a sense of purpose and meaning in their work (Aboobaker et al., 2020). In the field of nursing, workplace spirituality can manifest as a connection to the broader mission of healthcare, a sense of contributing to societal wellbeing, and experiencing personal growth and fulfillment through caregiving.

Work-life balance, as elucidated by Dewi et al. (2020), involves more than just dividing time between work and personal life. It encompasses achieving psychological equilibrium and satisfaction in both areas. In nursing, where the demands of the profession can be intense and all-consuming, achieving this balance is crucial for personal wellbeing and professional effectiveness.

Several studies support this hypothesis. For instance, research by Sony and Mekoth (2019) indicates that employees who experience high levels of workplace spirituality often report better work-life balance. This is particularly relevant in nursing, where the spiritual aspects of caregiving can provide a sense of accomplishment and personal growth, contributing to a more balanced and satisfying life overall (Hafni et al., 2020).

Given the profound influence of workplace spirituality in fostering a harmonious balance between professional and personal domains, the following hypothesis is presented:

**Hypothesis 3 (H3):** The presence of workplace spirituality will significantly enhance the work-life balance experienced by nurses.

Empirical research has underscored the combined influence of workplace spirituality and work-life balance on perceived professional benefits. Sony and Mekoth (2019) found that employees who reported high levels of both workplace spirituality and work-life balance exhibited greater job satisfaction, career advancement opportunities, and overall professional wellbeing. Similarly, Hafni et al. (2020) discovered that employees who are spiritually aligned with their work and maintain a balance between personal and professional life are more likely to perceive their job as beneficial and rewarding. Additionally, Garg et al. (2019) noted that individuals who achieve spiritual fulfillment at work and a balanced personal life are more engaged, committed, and invested in their professional roles, thereby enhancing their perceived benefits.

According to Aboobaker et al. (2020), when individuals resonate deeply with the spiritual and meaningful aspects of their work, they typically view their job as more valuable and beneficial. Conversely, as elucidated by Zhang (2020), a well-managed work-life balance ensures that professional responsibilities do not overshadow personal wellbeing. Maintaining this balance enables individuals to excel in their professional roles, leading to increased recognition, satisfaction, and perceived benefits (Rezapouraghdam et al., 2019).

Given the intertwined influence of workplace spirituality and work-life balance in shaping the perceived professional benefits, the following hypothesis is presented:

**Hypothesis 4 (H4):** The combined presence of workplace spirituality will significantly enhance the perceived professional benefits experienced by nurses.

**Hypothesis 5 (H5):** The combined presence of work-life balance will significantly enhance the perceived professional benefits experienced by nurses.

### 2.5 Mediating roles of workplace spirituality and work-life balance

Mindfulness is characterized by heightened awareness and presence, aimed at enhancing one’s connection to their work, thereby fostering workplace spirituality. Research by Petchsawang and Duchon (2012) suggests that mindfulness practices can lead to a deeper sense of purpose and fulfillment in one’s job, which are key components of workplace spirituality. This enhanced sense of spiritual connection at work could, in turn, lead to greater perceived professional benefits, as employees who find deeper meaning and purpose in their work are likely to experience higher job satisfaction and engagement (Saks, 2011). Workplace spirituality, as defined by researchers like Milliman et al. (2003), includes a sense of meaningful work, interconnectedness with others, and alignment with organizational values. This concept is increasingly recognized as a crucial factor in enhancing job satisfaction and overall wellbeing in the workplace.

The concept of work-life balance, as proposed by Greenhaus et al. (2003), refers to the extent to which an individual is equally engaged in and equally satisfied with their work role and family role. Studies have shown that mindfulness can reduce work-related stress and improve coping strategies, which are essential for maintaining a healthy work-life balance (Allen and Kiburz, 2012). By enhancing an individual’s ability to stay present and engaged in the moment, mindfulness can help mitigate the spillover of stress and anxiety from work to personal life, thereby promoting a more harmonious balance (Crain et al., 2017). This improved work-life balance is likely to lead to enhanced perceived professional benefits, as employees who successfully manage their work and personal life tend to report higher job satisfaction (Haar et al., 2014; Kasbuntoro et al., 2020).

Given that mindfulness has been previously proposed in this study to bolster both workplace spirituality and work-life balance, it is plausible that the linkage between mindfulness and perceived professional benefits can be mediated by these two pivotal aspects. Thus, the subsequent set of hypotheses is presented as follows:

**Hypothesis 6 (H6):** The positive relationship between Mindfulness and Perceived Professional Benefits will be mediated by Workplace Spirituality.

**Hypothesis 7 (H7):** The positive relationship between Mindfulness and Perceived Professional Benefits will be mediated by Work-Life Balance.

## 3 Materials and methods

### 3.1 Sample and data collection procedure

This study employed snowball and convenience sampling methods. Initially, the researchers contacted head nurses from five hospitals, who assisted in distributing the survey questionnaires to the nurses. Through these initial contacts, the researchers were introduced to more head nurses across various hospital departments. All participants were briefly informed about the purpose of the survey, and it was emphasized that their participation was anonymous and the data would be used solely for academic research. Additionally, as a token of appreciation, each nurse who participated in the survey received a coffee coupon.

From September to November 2023, 500 questionnaires were distributed to nurses across eight hospitals in Jiangxi Province. After excluding invalid responses, a total of 303 valid questionnaires were collected, resulting in a response rate of 60.6%. Table 1 presents the demographic characteristics of the 303 nurses who participated in the survey. The respondents’ characteristics were as follows:(1) In terms of age, the majority falls within the 22–32 age bracket, accounting for 35.64%, followed by the 33–42 age group at 33.99%. (2) Looking at gender distribution, there were 55 male respondents and 248 female respondents, indicating a higher proportion of female participants. (3) Regarding educational background, respondents who graduated from high school or vocational schools accounted for 36.63%, while those with a bachelor’s degree constituted the highest percentage at 56.44%. Respondents with postgraduate or higher educational qualifications accounted for only 3.3%. (4) Concerning income, 39.27% of respondents reported a monthly income ranging between 4,000 to 6,000 Chinese Yuan (approximately 559 to 838 US dollars), while 38.94% indicated a monthly income between 6,001 to 8,000 Chinese Yuan (approximately 838 to 1,118 US dollars) The demographic information of this survey closely aligns with the data published in the ‘China Health Statistics Yearbook,’ suggesting that the sample is representative.

**TABLE 1:** Participate profile (*N* = 303).

Demographic factor	Descriptive statistics
Age	22–32:108 (35.64%)
	33–42:103 (33.99%)
	43–55:92 (30.36%)
Gender	Male: 55 (18.15%)
	Female: 248 (81.85%)
Education	Below high school: 11 (3.63%)
	High school/vocational school: 111 (36.63%)
	College/University: 171 (56.44%)
	Master or higher: 10 (3.3%)
Monthly salary	Less than 4000 CNY: 41 (13.53%)
	4001–6000 CNY: 119 (39.27%)
	6001–8000 CNY: 118 (38.94%)
	More than 8000 CNY: 25 (8.25%)

### 3.2 Measurements

Trait mindfulness is measured on the mindfulness attention and awareness scale (MAAS) developed by Brown and Ryan (2003). The scale contains 15 questions. Sample items are “I find it difficult to stay focused on what’s happening in the present” and “I rush through activities without being really attentive to them.” All questions in the original scale were scored on a five-point Likert-Scale ranging from 1 = almost always to 5 = almost never. A low score represents a low level of trait mindfulness, a high score a high level of trait mindfulness.

Workplace spirituality in this study primarily focuses on its association with employees’ work attitudes and behaviors. Consequently, this study adopted three dimensions related to employee workplace behaviors from the scale developed by Ashmos and Duchon (2000) and Milliman et al. (2003): sense of work meaning, team connectedness, and alignment with organizational values. The scale for assessing workplace spirituality in this study comprised 8 items. Sample items include “I enjoy and am passionate about my work,” “I have a close connection with my colleagues,” and “I resonate with the mission and values of the organization.” Responses were gauged using a five-point Likert scale. The overall internal consistency for the workplace spirituality scale was found to be satisfactory (α = 0.892).

Work–life balance was measured by the scale developed by Shukla and Srivastava (2016), which has four items. These items were rated on five-point Likert scale ranging from 1 (strongly disagree) to 5 (strongly agree). Sample items are “I am able to balance between time at work and time at other activities” and “I feel that the job and other activities are currently balanced.” The reliability of this scale was satisfactory (α = 0.813).

Perceived Professional Benefits Scale (PPBS). This scale was adapted from the Nurse’s Perceived Professional Benefits Scale originally developed by Yanli et al. (2019), tailored to be applicable for eldercare nurses. The scale comprises six items, encompassing five dimensions: personal growth, positive nurse-patient relationships, recognition from family and friends, positive professional perception, and a sense of team belonging. Each item is rated using a five-point Likert scale, with scores ranging from 1 (strongly disagree) to 5 (strongly agree).

Control variables, including age, gender, and nursing experience, were incorporated in the analysis. Age was measured in years; gender was measured as a dummy variable in which male was coded as 1 and female was coded as 0, and nursing experience was measured by the number of years that respondents had worked in the nursing profession.

### 3.3 Data analysis

The model estimation is performed using partial least squares structural equation modeling (PLS-SEM). PLS-SEM requires lesser statistical specifications than the covariance based SEM (Chin, 1998). In particular, using PLS-SEM provides less bias estimation when the data are not normally distributed (Hair et al., 2011). PLS-SEM is an appropriate estimation method for this study given that the results from the Jarque-Bera test of normality indicate that all variables in the model do not follow abnormal distribution pattern. Hair et al. (2017) also recommend using PLS-SEM when analyzing complex models. This is suitable for the model proposed in this study that contains the measure of mindfulness that has 15 indicators, and the analysis of the mediating effect of workplace spirituality and work-life balance. Warp PLS 7.0 is used to perform the PLS-SEM estimation.

## 4 Result

### 4.1 Reliability and validity analysis

The internal consistency of the scales was determined using Cronbach’s alpha and composite reliability coefficients (Nunnally, 1978). As shown in Table 2, the Cronbach’s alpha for all variables ranged between 0.835 and 0.934, and the composite reliability coefficients were all above the recommended minimum value of 0.70 (Hair et al., 2011), indicating good internal consistency of the scales.

**TABLE 2:** Reliability and validity testing.

Variable	Cronbach’s alpha	Composite reliability coefficient	AVE	CR
MFN	0.934	0.938	0.521	0.938
WS	0.857	0.901	0.535	0.901
WLB	0.835	0.833	0.555	0.833
PPB	0.902	0.904	0.611	0.904

MFN, mindfulness; WS, workplace spirituality; PPB, perceived professional benefits; WLB, work-life balance; AVE, average variance extracted; CR, composite reliability.

Average Variance Extracted (AVE) and Composite Reliability (CR) were used for assessing convergent validity. Typically, an AVE value greater than 0.5 and a CR value greater than 0.7 indicate high convergent validity (Fornell and Larcker, 1981). In this study, a Confirmatory Factor Analysis (CFA) was conducted for four factors and 33 items. As can be seen from Table 2, the AVE values for all four factors were above 0.5, and the CR values were all above 0.7, suggesting that the data has good convergent validity. This confirms the acceptable convergent validity of all variables.

In Table 3, the diagonal values represent the square roots of the Average Variance Extracted (AVE), while the other values are correlation coefficients. For discriminant validity analysis, the square root of the AVE for the Mindfulness factor (MFN) is 0.722, which exceeds the maximum absolute value of the inter-factor correlation coefficients, 0.401, indicating good discriminant validity. For Workplace Spirituality (WS), the square root of the AVE is 0.731, surpassing the maximum absolute value of the inter-factor correlation coefficients, 0.417, thus demonstrating good discriminant validity. For Work-Life Balance (WLB), the square root of the AVE is 0.745, greater than the maximum absolute value of the inter-factor correlation coefficients, 0.503, signifying good discriminant validity. For Perceived Professional Benefits (PPB), the square root of the AVE is 0.781, higher than the maximum absolute value of the inter-factor correlation coefficients, 0.503, suggesting good discriminant validity. As illustrated in Table 3, these findings indicate that the variables in this study exhibit good discriminant validity.

**TABLE 3:** Discriminant validity testing.

Variables	MFN	WS	WLB	PPB
MFN	(0.722)			
WS	0.381**	(0.731)		
WLB	0.384**	0.417**	(0.745)	
PPB	0.401**	0.412**	0.503**	(0.781)

Square roots of average variance extracted are show on the diagonal; off-diagonal values are Pearson correlation coefficients.

**p < 0.01.

### 4.2 Hypothesis testing

This study conducted analyses to examine potential issues of multicollinearity and Common Method Bias (CMB) that might affect the estimates. Multicollinearity was checked by confirming that the Variance Inflation Factor (VIF) did not exceed 3.3, which would indicate the presence of multicollinearity (Petter et al., 2007). The results affirmed that the full VIF statistics in the model ranged from 1.206 to 1.252, thus not surpassing the maximum threshold of 3.3, thus, multicollinearity is not a serious issue. Additionally, in this study, the structural model fit indices, including χ^2^/df = 1.104, GFI = 0.914, AGFI = 0.901, NFI = 0.921, CFI = 0.981, TLI = 0.980, and RMSEA = 0.027, indicate a good fit with the data. Concurrently, based on Table 3, the correlations between the variables were examined. Significant correlations among the independent variables, mediating variables, and dependent variables provide preliminary support for all hypotheses.

**FIGURE 1:**
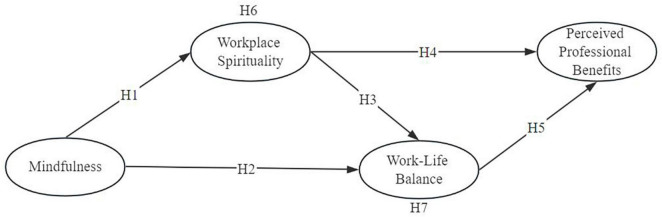
The hypothesized model.

**FIGURE 2:**
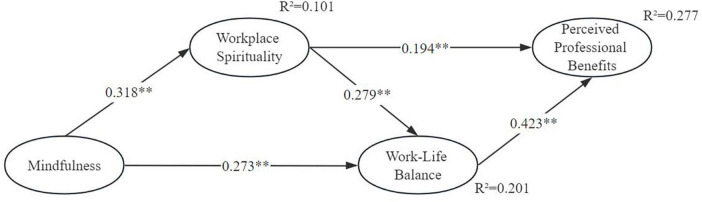
Results from hypotheses testing.

The results from hypotheses are presented in Figures 1, 2 and Table 4. Hypothesis 1 states that there is a positive relationship between mindfulness and workplace spirituality (β = 0.318; *p* < 0.001), thus, hypothesis 1 was supported. Hypothesis 2 states that mindfulness is positively related with work-life balance (β = 0.273; *p* < 0.001), therefore, hypothesis 2 is supported. Hypothesis 3 states that workplace spirituality is positively related with work-life balance (β = 0.279; *p* < 0.001), hypothesis 3 is supported. Hypothesis 4 states that workplace spirituality is positively related with perceived professional benefits (β = 0.194; *p* < 0.001), hypothesis 4 is supported. Hypothesis 5 states that work–life balance is positively related with perceived professional benefits (β = 0.423; *p* < 0.001), hypothesis 5 is supported.

**TABLE 4:** Standardized indirect effects.

	Point estimate	Product of coefficients		Bootstrapping		
				**Percentile 95% CI**		**Two-tailed significance**
		**SE**	**Z**	**Lower**	**Upper**	
MFN = > WS = > PPB	0.157	0.028	5.621	0.100	0.208	0.000***
MFN = > WLB = > PPB	0.086	0.021	4.061	0.044	0.128	0.000***

Standardized estimations of 5000 bootstrap samples.

***p < 0.001. MFN, mindfulness; WS, workplace spirituality; WLB, work-life balance; PPB, perceived professional benefits.

The study hypothesized that mindfulness affects perceived professional benefits through two mediators: workplace spirituality and work–life balance. To test for mediating effects, the researchers used the bootstrap method (Bollen and Stine, 1990). The results of the 95% confidence intervals of the 5,000 bootstrap samples are shown in Table 3. The absolute values of all Z values are significant (greater than 1.96), and the 95% confidence intervals do not include zero. Hypothesis 6 states that workplace spirituality mediates the positive relationship between mindfulness and perceived professional benefits (standardized indirect effect = 0.086, *p* < 0.001). Therefore, Hypothesis 6 is supported. Additionally, for Hypothesis 7, work–life balance significantly mediated the relationship between mindfulness and perceived professional benefits (standardized indirect effect = 0.157, *p* < 0.001), supporting Hypothesis 7.

## 5 Discussion

### 5.1 Theoretical implications

This study extends the understanding of mindfulness in the workplace context, aligning with Cognitive Resource Theory. It demonstrates how mindfulness, as a cognitive resource, positively impacts workplace spirituality and work-life balance, thus enhancing perceived professional benefits in high-stress environments like nursing.

Firstly, by linking mindfulness to workplace spirituality and work-life balance, this study applies Cognitive Resource Theory to a new domain. It suggests that mindfulness, as a cognitive resource, enables nurses to better align with their work’s spiritual aspects, enhancing job satisfaction and organizational commitment. This aligns with previous research by Shapiro et al. (2008) and Goodman and Schorling (2012), which highlighted the benefits of mindfulness in enhancing personal wellbeing and professional engagement. Our findings suggest that mindfulness is beneficial not only for personal wellbeing but also plays a crucial role in fostering a sense of spiritual connection and balance in the workplace.

Moreover, the study contributes to the growing body of research on workplace spirituality, a relatively underexplored area in nursing. By establishing a positive link between mindfulness and workplace spirituality, our research underscores the importance of spiritual wellbeing in high-stress professions like nursing. This finding resonates with the work of Altaf and Awan (2011), who emphasized the relevance of workplace spirituality in enhancing job satisfaction and organizational commitment.

Another significant contribution of this study is the exploration of the relationship between work-life balance and perceived professional benefits. Our results indicate that nurses who experience a better work-life balance are likely to perceive higher professional benefits. This finding adds to the literature on work-life balance, supporting the theories proposed by Sirgy and Lee (2018), which emphasize the importance of achieving equilibrium between work and personal life for professional success and satisfaction.

Importantly, our study also highlights the mediating roles of workplace spirituality and work-life balance in the relationship between mindfulness and perceived professional benefits. This nuanced understanding of the interplay between these variables provides a more comprehensive view of how mindfulness can influence professional outcomes in high-stress environments. This aspect of our research offers a new perspective, contributing to the theoretical development in the fields of mindfulness and occupational wellbeing.

In summary, the inclusion of Cognitive Resource Theory in this study provides a richer theoretical framework for understanding the role of mindfulness in the workplace. It not only reinforces existing theories but also offers new insights into how cognitive resources like mindfulness can be optimized in high-pressure work environments to enhance professional wellbeing and performance.

### 5.2 Practical implications

The findings of this study offer several practical implications for the nursing profession, particularly in high-stress environments such as hospitals. These implications are relevant for healthcare administrators, nursing educators, and nurses themselves, aiming to enhance workplace wellbeing and professional efficacy. Firstly, the demonstrated positive relationship between mindfulness and workplace spirituality suggests that incorporating mindfulness training into the professional development programs for nurses could be beneficial. Mindfulness practices can help nurses cultivate a deeper sense of connection to their work, enhancing their sense of purpose and fulfillment. This, in turn, can lead to improved job satisfaction and reduced burnout, which are crucial in high-stress healthcare settings. Secondly, the study highlights the importance of work-life balance in enhancing perceived professional benefits among nurses. Healthcare institutions should consider implementing policies and programs that support a healthy work-life balance. This could include flexible scheduling, wellness programs, and resources for stress management. By prioritizing work-life balance, hospitals can improve job satisfaction and retention rates among nurses.

Moreover, the finding that workplace spirituality and work-life balance mediate the relationship between mindfulness and perceived professional benefits underscores the need for a holistic approach to nurse wellbeing. Healthcare organizations could benefit from creating a work environment that nurtures spiritual wellbeing and allows for personal life balance. This could involve creating spaces for reflection, encouraging team-building activities that foster a sense of community, and recognizing the individual needs of nurses. Additionally, Because state mindfulness is not a quality that some individuals possess and others lack, this suggests that mindfulness training should not be viewed as a one-time intervention but as an ongoing practice. Regular mindfulness sessions, whether through guided meditation, mindful breathing exercises, or other techniques, can be integrated into the nurses’ routine, potentially leading to long-term improvements in their professional and personal lives. Finally, the study’s findings can inform nursing education and training. Incorporating mindfulness and stress management techniques into nursing curricula can prepare future nurses to better handle the demands of their profession. This could involve practical workshops, simulation exercises, and coursework that emphasize the development of mindfulness and its application in clinical settings.

### 5.3 Limitations of the study

Despite the contributions of this study, some limitations exist that must be addressed. Firstly, the study’s findings are based on data collected from nurses in eight hospitals in Jiangxi Province, China. This geographical and institutional specificity may limit the generalizability of the results. The context-specific nature of the findings suggests caution when applying these results to different nursing environments or cultural settings. Secondly, our study utilized self-reported measures to assess mindfulness, workplace spirituality, work-life balance, and perceived professional benefits. While self-reporting is a prevalent method in psychological research, it is subject to biases such as social desirability and self-perception discrepancies. This reliance on subjective measures may affect the accuracy and objectivity of the findings. Thirdly, the cross-sectional design of this study limits the ability to infer causality among the studied variables. This design constraint means that the study provides a snapshot of the relationships at a single point in time, without accounting for changes or developments over time. Fourthly, while the study identified workplace spirituality and work-life balance as mediators, the specific underlying mechanisms of these mediating effects were not empirically tested. This gap leaves room for further exploration into how these mediating factors operate within the context of mindfulness and perceived professional benefits.

### 5.4 Future research

Firstly, future research could expand the scope by including nurses from various regions and healthcare settings. This expansion would enhance the applicability of the findings across different nursing environments and potentially provide a more comprehensive understanding of the studied phenomena. Secondly, to validate and enrich the findings, future studies might consider incorporating more objective measures. Additionally, triangulating data with qualitative interviews or observations could provide a more nuanced understanding of the relationships among mindfulness, workplace spirituality, work-life balance, and perceived professional benefits. Thirdly, implementing longitudinal or experimental designs in future research could provide stronger evidence of causal relationships. These designs would allow for a deeper understanding of the dynamics of these variables over time, particularly in high-stress nursing environments. Fourthly, future research should explore the specific pathways through which mindfulness influences workplace spirituality and work-life balance. Additionally, studies could investigate the impact of mindfulness-based interventions in the workplace, providing practical insights into how mindfulness training can be effectively integrated into nursing practice to enhance professional wellbeing and patient care. Lastly, considering the evolving nature of the nursing profession and the increasing emphasis on mental wellbeing, future studies could adapt their focus to align with these changes. This adaptation could involve exploring new areas of mindfulness application or addressing emerging challenges in the nursing profession.

## 6 Conclusion

This study significantly contributes to understanding the role of mindfulness in enhancing workplace spirituality, work-life balance, and perceived professional benefits among nurses in high-stress environments. The positive correlations identified between mindfulness and both workplace spirituality and work-life balance, and their impact on professional benefits, highlight the importance of mindfulness in the nursing profession. These findings suggest that integrating mindfulness training into nursing practices and professional development could lead to improved job satisfaction, reduced burnout, and enhanced overall wellbeing. The research underscores the need for a holistic approach in healthcare settings, emphasizing the creation of supportive environments that foster spiritual wellbeing and work-life balance. While the study is limited by its regional focus and reliance on self-reported data, it opens avenues for future research to explore these relationships further and in more diverse contexts. Overall, the study advocates for mindfulness as a key component in healthcare strategies to improve the efficacy and wellbeing of nurses in demanding work environments.

## Data availability statement

The original contributions presented in the study are included in the article/supplementary material, further inquiries can be directed to the corresponding author.

## Ethics statement

Ethical review and approval was not required for the study on human participants in accordance with the local legislation and institutional requirements. Written informed consent from the patients/participants or patients/participants’ legal guardian/next of kin was not required to participate in this study in accordance with the national legislation and the institutional requirements.

## Author contributions

YL: Conceptualization, Formal analysis, Funding acquisition, Resources, Writing – original draft, Writing – review and editing. CJ: Formal analysis, Funding acquisition, Investigation, Resources, Validation, Visualization, Writing – original draft, Writing – review and editing. YP: Data curation, Formal analysis, Investigation, Methodology, Resources, Visualization, Writing – original draft, Writing – review and editing. ZX: Conceptualization, Investigation, Methodology, Project administration, Resources, Validation, Visualization, Writing – original draft, Writing – review and editing.

## References

[B1] Aboobaker N. Edward M. Ka Z. (2020). Workplace spirituality and employee loyalty: An empirical investigation among millennials in India. *J. Asia Bus. Stud.* 14 211–225. 10.1108/JABS-03-2018-0089

[B2] Allen T. D. Kiburz K. M. (2012). Trait mindfulness and work–family balance among working parents: The mediating effects of vitality and sleep quality. *J. Vocat. Behav.* 80 372–379. 10.1016/j.jvb.2011.09.002

[B3] Altaf A. Awan M. A. (2011). Moderating affect of workplace spirituality on the relationship of job overload and job satisfaction. *J. Bus. Ethics* 104 93–99. 10.1007/s10551-011-0891-0

[B4] Althammer S. E. Reis D. Van der Beek S. Beck L. Michel A. (2021). A mindfulness intervention promoting work–life balance: How segmentation preference affects changes in detachment, well-being, and work–life balance. *J. Occupat. Organ. Psychol.* 94 282–308. 10.1111/joop.12346

[B5] Ashmos D. P. Duchon D. (2000). Spirituality at work: A conceptualization and measure. *J. Manag. Inq.* 9 134–145. 10.1177/105649260092008

[B6] Baer R. A. Smith G. T. Allen K. B. (2004). Assessment of mindfulness by self-report: The Kentucky inventory of mindfulness skills. *Assessment* 11 191–206. 10.1177/1073191104268029 15358875

[B7] Bakhamis L. Paul I. D. P. Smith H. Coustasse A. (2019). Still an epidemic: The burnout syndrome in hospital registered nurses. *Health Care Manage.* 38 3–10. 10.1097/HCM.0000000000000243 30640239

[B8] Bamber M. D. Schneider J. K. (2022). College students’ perceptions of mindfulness-based interventions: A narrative review of the qualitative research. *Curr. Psychol.* 41 667–680. 10.1007/s12144-019-00592-4

[B9] Bishop S. R. Lau M. Shapiro S. Carlson L. Anderson N. D. Carmody J. (2004). Mindfulness: A proposed operational definition. *Clin Psychol.* 11 230–241. 10.1093/clipsy.bph077

[B10] Bollen K. A. Stine R. (1990). Direct and indirect effects: Classical and bootstrap estimates of variability. *Sociol. Methodol.* 20 115–140. 10.2307/271084

[B11] Brown K. W. Ryan R. M. (2003). The benefits of being present: Mindfulness and its role in psychological well-being. *J. Pers. Soc. Psychol.* 84:822. 10.1037/0022-3514.84.4.822 12703651

[B12] Charoensukmongkol P. Puyod J. V. (2022). Mindfulness and emotional exhaustion in call center agents in the Philippines: Moderating roles of work and personal characteristics. *J. General Psychol.* 149 72–96. 10.1080/00221309.2020.1800582 32748707

[B13] Chen S. Murphy D. (2019). The mediating role of authenticity on mindfulness and wellbeing: A cross cultural analysis. *Asia Pac. J. Counsell. Psychother.* 10 40–55. 10.1080/21507686.2018.1556171

[B14] Chin W. W. (1998). The partial least squares approach to structural equation modeling. *Modern Methods Bus. Res.* 295, 295–336.

[B15] Christianson K. L. (2020). Emotional intelligence and critical thinking in nursing students: Integrative review of literature. *Nurse Educ.* 45 E62–E65. 10.1097/NNE.0000000000000801 32091477

[B16] Crain T. L. Schonert-Reichl K. A. Roeser R. W. (2017). Cultivating teacher mindfulness: Effects of a randomized controlled trial on work, home, and sleep outcomes. *J. Occupat. Health Psychol.* 22:138. 10.1037/ocp0000043 27182765

[B17] Dane E. (2011). Paying attention to mindfulness and its effects on task performance in the workplace. *J. Manage.* 37 997–1018. 10.1177/0149206310367948

[B18] Daniel C. Gentina E. Mesmer-Magnus J. (2022). Mindfulness buffers the deleterious effects of workaholism for work-family conflict. *Soc. Sci. Med.* 306:115118. 10.1016/j.socscimed.2022.115118 35696778

[B19] Davidson R. J. Kabat-Zinn J. Schumacher J. Rosenkranz M. Muller D. Santorelli S. F. (2003). Alterations in brain and immune function produced by mindfulness meditation. *Psychosom. Med.* 65 564–570. 10.1097/01.PSY.0000077505.67574.E3 12883106

[B20] Delgado C. Upton D. Ranse K. Furness T. Foster K. (2017). Nurses’ resilience and the emotional labour of nursing work: An integrative review of empirical literature. *Int. J. Nurs. Stud.* 70 71–88. 10.1016/j.ijnurstu.2017.02.008 28235694

[B21] Dewi S. S. Madjid A. Fauzan A. (2020). The role of religiosity in work-life balance. *Budapest Int. Res. Crit. Inst.* 3 2363–2374. 10.33258/birci.v3i3.1192

[B22] Eby L. T. Robertson M. M. Facteau D. B. (2020). “Mindfulness and relationships: An organizational perspective,” in *Research in Personnel and Human Resources Management*, eds Ronald Buckley M. Wheeler A. R. Halbesleben J. R. B. (Bingley: Emerald Publishing Limited), 57–102.

[B23] Fiedler F. E. (1989). Cognitive resource theory and leadership in organizations. *J. Appl. Psychol.* 74 802–814. 10.1037/0021-9010.74.5.802

[B24] Fornell C. Larcker D. F. (1981). Evaluating structural equation models with unobservable variables and measurement error. *J. Mark. Res.* 18 39–50. 10.2307/3151312

[B25] Foster S. Foster A. (2019). The impact of workplace spirituality on work-based learners: Individual and organisational level perspectives. *J. Work Appl. Manage.* 11 63–75. 10.1108/JWAM-06-2019-0015

[B26] Garg N. Punia B. K. Jain A. (2019). Workplace spirituality and job satisfaction: Exploring mediating effect of organization citizenship behaviour. *Vision* 23 287–296. 10.1177/0972262919850928

[B27] Garland E. L. Farb N. A. Goldin P. Fredrickson B. L. (2015). Mindfulness broadens awareness and builds eudaimonic meaning: A process model of mindful positive emotion regulation. *Psychol. Inq.* 26 293–314. 10.1080/1047840X.2015.1064294 27087765 PMC4826727

[B28] Good D. J. Lyddy C. J. Glomb T. M. Bono J. E. Brown K. W. Duffy M. K. (2016). Contemplating mindfulness at work: An integrative review. *J. Manage.* 42 114–142. 10.1177/0149206315617003

[B29] Goodman M. J. Schorling J. B. (2012). A mindfulness course decreases burnout and improves well-being among healthcare providers. *Int. J. Psychiatry Med.* 43 119–128. 10.2190/PM.43.2.b 22849035

[B30] Greenhaus J. H. Collins K. M. Shaw J. D. (2003). The relation between work–family balance and quality of life. *J. Vocat. Behav.* 63 510–531. 10.1016/S0001-8791(02)00042-8

[B31] Haar J. M. Russo M. Suñe A. Ollier-Malaterre A. (2014). Outcomes of work–life balance on job satisfaction, life satisfaction and mental health: A study across seven cultures. *J. Vocat. Behav.* 85 361–373. 10.1016/j.jvb.2014.08.010

[B32] Hafenbrack A. C. Vohs K. D. (2018). Mindfulness meditation impairs task motivation but not performance. *Organ. Behav. Hum. Decis. Process.* 147 1–15. 10.1016/j.obhdp.2018.05.001

[B33] Hafni L. Budiyanto B. Suhermin S. Chandra T. Priyono P. (2020). The role of workplace spirituality in improving job satisfaction and lecturer performance. *J. Talent Dev. Excell.* 12 1262–1282.

[B34] Hair J. Hollingsworth C. L. Randolph A. B. Chong A. Y. L. (2017). An updated and expanded assessment of PLS-SEM in information systems research. *Indust. Manage. Data Syst.* 117 442–458. 10.1108/IMDS-04-2016-0130

[B35] Hair J. F. Ringle C. M. Sarstedt M. (2011). PLS-SEM: Indeed a silver bullet. *J. Mark. Theory Pract.* 19 139–152. 10.2753/MTP1069-6679190202

[B36] Hölzel B. K. Lazar S. W. Gard T. Schuman-Olivier Z. Vago D. R. Ott U. (2011). How does mindfulness meditation work? Proposing mechanisms of action from a conceptual and neural perspective. *Perspect. Psychol. Sci.* 6 537–559. 10.1177/1745691611419671 26168376

[B37] Huang J. Liu F. Teng Z. Chen J. Zhao J. Wang X. (2020). Care for the psychological status of frontline medical staff fighting against coronavirus disease 2019 (COVID-19). *Clin. Infect. Dis.* 71 3268–3269. 10.1093/cid/ciaa385 32246142 PMC7184370

[B38] Indradevi R. (2020). Workplace spirituality: Successful mantra for modern organization. *J. Crit. Rev.* 7 437–440. 10.31838/jcr.07.06.77

[B39] Jha A. P. Krompinger J. Baime M. J. (2007). Mindfulness training modifies subsystems of attention. *Cognit. Affect. Behav. Neurosci.* 7 109–119. 10.3758/CABN.7.2.109 17672382

[B40] Jnaneswar K. Sulphey M. (2021). A study on the relationship between workplace spirituality, mental wellbeing and mindfulness. *Manag. Sci. Lett.* 11 1045–1054. 10.5267/j.msl.2020.9.038

[B41] Kabat-Zinn J. (1990). *Full Catastrophe Living: Using the Wisdom of Your Body and Mind to Face Stress, Pain, and Illness.* New York, NY: Bantam Dell.

[B42] Kabat-Zinn J. (1994). *Wherever you Go, there you are: Mindfulness Meditation in Everyday Life.* New York, NY: Hyperion.

[B43] Kasbuntoro D. I. Maemunah S. Mahfud I. Fahlevi M. Parashakti R. D. (2020). Work-life balance and job satisfaction: A case study of employees on banking companies in Jakarta. *Int. J. Control Autom.* 13 439–451.

[B44] Khamisa N. Oldenburg B. Peltzer K. Ilic D. (2015). Work related stress, burnout, job satisfaction and general health of nurses. *Int. J. Environ. Res. Public Health* 12 652–666. 10.3390/ijerph120100652 25588157 PMC4306884

[B45] King E. Haar J. M. (2017). Mindfulness and job performance: A study of Australian leaders. *Asia Pac. J. Hum. Resour.* 55 298–319. 10.1111/1744-7941.12143

[B46] Kirca N. Bademli K. (2019). Relationship between communication skills and care behaviors of nurses. *Perspect. Psychiatr. Care* 55 624–631. 10.1111/ppc.12381 30990906

[B47] Kudesia R. S. (2019). Mindfulness as metacognitive practice. *Acad. Manage. Learn. Educ.* 18 286–304. 10.5465/amle.2017.0351

[B48] Levett K. M. Coughlan S. Longridge S. Roumeliotis V. Adams J. (2019). Be well: A systems-based wellness intervention using mindfulness in the workplace–A case study. *J. Manage. Organ.* 25 613–634. 10.1017/jmo.2017.41

[B49] Liu S. Xin H. Shen L. He J. Liu J. (2020). The influence of individual and team mindfulness on work engagement. *Front. Psychol.* 10:2928. 10.3389/fpsyg.2019.02928 32038356 PMC6985205

[B50] Lutz A. Jha A. P. Dunne J. D. Saron C. D. (2016). Investigating the phenomenological matrix of mindfulness-related practices from a neurocognitive perspective. *Am. Psychol.* 70 632–658. 10.1037/a0039585 26436313 PMC4608430

[B51] McNall L. A. Tombari J. M. Brown M. M. (2021). Exploring how mindfulness links to work outcomes: Positive affectivity and work-life enrichment. *Appl. Res. Qual. Life* 16 167–182. 10.1007/s11482-019-09762-9

[B52] Mellner C. Osika W. Niemi M. (2022). Mindfulness practice improves managers’ job demands-resources, psychological detachment, work-nonwork boundary control, and work-life balance–A randomized controlled trial. *Int. J. Workplace Health Manage.* 15 493–514. 10.1108/IJWHM-07-2021-0146

[B53] Milliman J. Czaplewski A. J. Ferguson J. (2003). Workplace spirituality and employee work attitudes: An exploratory empirical assessment. *J. Organ. Change Manage.* 16 426–447. 10.1108/09534810310484172

[B54] Murphy S. E. Hooijberg R. Kickul J. (1992). Cognitive resource theory and the utilization of the leader’s and group members’ technical competence. *Leadersh. Q.* 3 237–255. 10.1016/1048-9843(92)90020-S

[B55] Nunnally J. C. (1978). “An overview of psychological measurement,” in *Clinical Diagnosis of Mental Disorders: A Handbook*, ed. Wolman B. B. (Boston, MA: Springer), 97–146. 10.1007/978-1-4684-2490-4_4

[B56] Onu C. A. Akinlabi B. H. Adegbola E. A. (2018). Work life balance and normative commitments of employees in the selected deposit money banks in Ogun State, Nigeria. *Eur. J. Bus. Innovat. Res.* 6 1–13. 10.37745/ejbir.2013

[B57] Pacheco T. Coulombe S. Meunier S. (2021). When work conflicts with personal projects: The association of work-life conflict with worker well being and the mediating role of mindfulness. *Front. Psychol.* 12:539582. 10.3389/fpsyg.2021.539582 34819891 PMC8606422

[B58] Pattanawit P. Charoensukmongkol P. (2022). Benefits of workplace spirituality on real estate agents’ work outcomes: The mediating role of person-job fit. *Manage. Res. Rev.* 45 1393–1411. 10.1108/MRR-06-2021-0482

[B59] Petchsawang P. Duchon D. (2012). Workplace spirituality, meditation, and work performance. *J. Manage. Spiritual. Relig.* 9 189–208. 10.1080/14766086.2012.688623

[B60] Petter S. Straub D. Rai A. (2007). Specifying formative constructs in information systems research. *MIS Q.* 31 623–656. 10.2307/25148814

[B61] Piotrowski A. Sygit-Kowalkowska E. Boe O. Rawat S. (2022). Resilience, occupational stress, job satisfaction, and intention to leave the organization among nurses and midwives during the COVID-19 pandemic. *Int. J. Environ. Res. Public Health* 19:6826. 10.3390/ijerph19116826 35682410 PMC9180178

[B62] Rathee R. Rajain P. (2020). Workplace spirituality: A comparative study of various models. *Jindal J. Bus. Res.* 9 27–40. 10.1177/2278682120908554

[B63] Reb J. Narayanan J. Chaturvedi S. (2014). Leading mindfully: Two studies on the influence of supervisor trait mindfulness on employee well-being and performance. *Mindfulness* 5 36–45. 10.1007/s12671-012-0144-z

[B64] Rehman M. U. Dastgeer G. (2022). Measuring employee’s level outcomes through mindfulness with mediating role of workplace spirituality. *Middle East J. Manage.* 9 574–588. 10.1504/MEJM.2022.10044166 35009967

[B65] Rezapouraghdam H. Alipour H. Arasli H. (2019). Workplace spirituality and organization sustainability: A theoretical perspective on hospitality employees’ sustainable behavior. *Environ. Dev. Sustain.* 21 1583–1601. 10.1007/s10668-018-0120-4

[B66] Roeser R. W. Skinner E. Beers J. Jennings P. A. (2012). Mindfulness training and teachers’ professional development: An emerging area of research and practice. *Child Dev. Perspect.* 6 167–173. 10.1111/j.1750-8606.2012.00238.x

[B67] Saks A. M. (2011). Workplace spirituality and employee engagement. *J. Manage. Spiritual. Relig.* 8 317–340. 10.1080/14766086.2011.630170

[B68] Salvarani V. Ardenghi S. Rampoldi G. Bani M. Cannata P. Ausili D. (2020). Predictors of psychological distress amongst nursing students: A multicenter cross-sectional study. *Nurse Educ. Pract.* 44:102758. 10.1016/j.nepr.2020.102758 32234667

[B69] Schluter P. J. Turner C. Huntington A. D. Bain C. J. McClure R. J. (2011). Work/life balance and health: The Nurses and Midwives e-cohort study. *Int. Nurs. Rev.* 58 28–36. 10.1111/j.1466-7657.2010.00849.x 21281290

[B70] Shapiro S. L. Oman D. Thoresen C. E. Plante T. G. Flinders T. (2008). Cultivating mindfulness: Effects on well-being. *J. Clin. Psychol.* 64 840–862. 10.1002/jclp.20491 18484600

[B71] Shukla A. Srivastava R. (2016). Development of short questionnaire to measure an extended set of role expectation conflict, coworker support and work-life balance: The new job stress scale. *Cogent Bus. Manage.* 3:1. 10.1080/23311975.2015.1134034

[B72] Siffleet J. Williams A. M. Rapley P. Slatyer S. (2015). Delivering best care and maintaining emotional wellbeing in the intensive care unit: The perspective of experienced nurses. *Appl. Nurs. Res.* 28 305–310. 10.1016/j.apnr.2015.02.008 26608430

[B73] Sirgy M. J. Lee D. J. (2018). Work-life balance: An integrative review. *Appl. Res. Qual. Life* 13 229–254. 10.1007/s11482-017-9509-8

[B74] Slutsky J. Chin B. Raye J. Creswell J. D. (2019). Mindfulness training improves employee well-being: A randomized controlled trial. *J. Occupat. Health Psychol.* 24:139. 10.1037/ocp0000132 30335419

[B75] Smith A. (2022). A holistic approach to the well-being of nurses: A combined effects approach. *Adv. Soc. Sci. Res. J.* 9 475–484. 10.14738/assrj.91.11650

[B76] Sony M. Mekoth N. (2019). The relationship between workplace spirituality, job satisfaction and job performance. *Int. J. Process Manage. Benchmark.* 9 27–46. 10.1504/IJPMB.2019.097819 35009967

[B77] Teper R. Segal Z. V. Inzlicht M. (2013). Inside the mindful mind: How mindfulness enhances emotion regulation through improvements in executive control. *Curr. Dir. Psychol. Sci.* 22 449–454. 10.1177/0963721413495869

[B78] Thimmapuram J. R. Grim R. Bell T. Benenson R. Lavallee M. Modi M. (2019). Factors influencing work–life balance in physicians and advance practice clinicians and the effect of heartfulness meditation conference on burnout. *Glob. Adv. Health Med.* 8:2164956118821056. 10.1177/2164956118821056 30733893 PMC6343441

[B79] Vecchio R. P. (1990). Theoretical and empirical examination of cognitive resource theory. *J. Appl. Psychol.* 75 141–157. 10.1037/0021-9010.75.2.141

[B80] Vermaak C. Görgens-Ekermans G. Nieuwenhuize C. (2017). Shift work, emotional labour and psychological well-being of nursing staff. *Management* 22 35–48. 10.30924/mjcmi/2017.22.2.35

[B81] Wheeler M. S. Arnkoff D. B. Glass C. R. (2017). The neuroscience of mindfulness: How mindfulness alters the brain and facilitates emotion regulation. *Mindfulness* 8 1471–1487.

[B82] Wu W. Zhang Y. Wang P. Zhang L. Wang G. Lei G. (2020). Psychological stress of medical staffs during outbreak of COVID-19 and adjustment strategy. *J. Med. Virol.* 92 1962–1970. 10.1002/jmv.25914 32314806 PMC7264502

[B83] Yang X. Jo W. (2022). Roles of work-life balance and trait mindfulness between recovery experiences and employee subjective well-being: A moderated mediation model. *J. Hosp. Tour. Manage.* 52 459–468. 10.1016/j.jhtm.2022.08.005

[B84] Yanli H. U. Jing H. U. Li L. Zhao B. Li F. (2019). Development and preliminary validation of a brief Nurses’ Perceived Professional Benefit Questionnaire (NPPBQ). *BMC Med. Res. Methodol.* 20:18. 10.21203/rs.2.11751/v1 PMC6993446 32000690

[B85] Zeidan F. Johnson S. K. Diamond B. J. David Z. Goolkasian P. (2010). Mindfulness meditation improves cognition: Evidence of brief mental training. *Conscious. Cogn.* 19 597–605. 10.1016/j.concog.2010.03.014 20363650

[B86] Zhang S. (2020). Workplace spirituality and unethical pro-organizational behavior: The mediating effect of job satisfaction. *J. Bus. Ethics* 161 687–705. 10.1007/s10551-018-3966-3

[B87] Zheng M. X. Masters-Waage T. C. Yao J. Lu Y. Tan N. Narayanan J. (2020). Stay mindful and carry on: Mindfulness neutralizes COVID-19 stressors on work engagement via sleep duration. *Front. Psychol.* 11:610156. 10.3389/fpsyg.2020.610156 33408674 PMC7779584

